# Clinical Applications of Exosomes: A Critical Review

**DOI:** 10.3390/ijms25147794

**Published:** 2024-07-16

**Authors:** Kar Wai Alvin Lee, Lisa Kwin Wah Chan, Lee Cheuk Hung, Lam Kar Wai Phoebe, Youngjin Park, Kyu-Ho Yi

**Affiliations:** 1 EverKeen Medical Centre, Hong Kong; alvin429@yahoo.com (K.W.A.L.); drchan.everkeen@gmail.com (L.K.W.C.); andylee618@hotmail.com (L.C.H.); 2 Perfect Skin Solution, Hong Kong; drlamkarwai@gmail.com; 3 Obliv Clinic, Incheon 21998, Republic of Korea; youngjinp@gmail.com; 4 Division in Anatomy and Developmental Biology, Department of Oral Biology, Human Identification Research Institute, BK21 FOUR Project, Yonsei University College of Dentistry, 50-1 Yonsei-ro, Seodaemun-gu, Seoul 03722, Republic of Korea; 5 Maylin Clinic (Apgujeong), Seoul B1F 450, Republic of Korea

**Keywords:** exosomes, therapeutic potential, regenerative medicine, cancer therapy, diagnostic biomarkers

## Abstract

Exosomes, small membrane-bound vesicles secreted by cells, have gained significant attention for their therapeutic potential. Measuring 30–100 nm in diameter and derived from various cell types, exosomes play a crucial role in intercellular communication by transferring proteins, lipids, and RNA between cells. This review analyzes existing literature on the clinical applications of exosomes. We conducted a comprehensive search of peer-reviewed articles and clinical trial data to evaluate the benefits, limitations, and challenges of exosome-based therapies. Key areas of focus included regenerative medicine, cancer therapy, gene therapy, and diagnostic biomarkers. This review highlights the vast clinical applications of exosomes. In regenerative medicine, exosomes facilitate tissue repair and regeneration. In cancer therapy, exosomes can deliver therapeutic agents directly to tumor cells. In gene therapy, exosomes serve as vectors for gene delivery. As diagnostic biomarkers, they are useful in diagnosing various diseases. Challenges such as the isolation, purification, and characterization of exosomes were identified. Current clinical trials demonstrate the potential of exosome-based therapies, though they also reveal significant hurdles. Regulatory issues, including the need for standardization and validation of exosome products, are critical for advancing these therapies. While significant progress has been made in understanding exosome biology, further research is essential to fully unlock their clinical potential. Addressing challenges in isolation, purification, and regulatory standardization is crucial for their successful application in clinical practice. This review provides a concise overview of the clinical applications of exosomes, emphasizing both their therapeutic promise and the obstacles that need to be overcome.

## 1. Comprehensive Background

**Figure 1:**
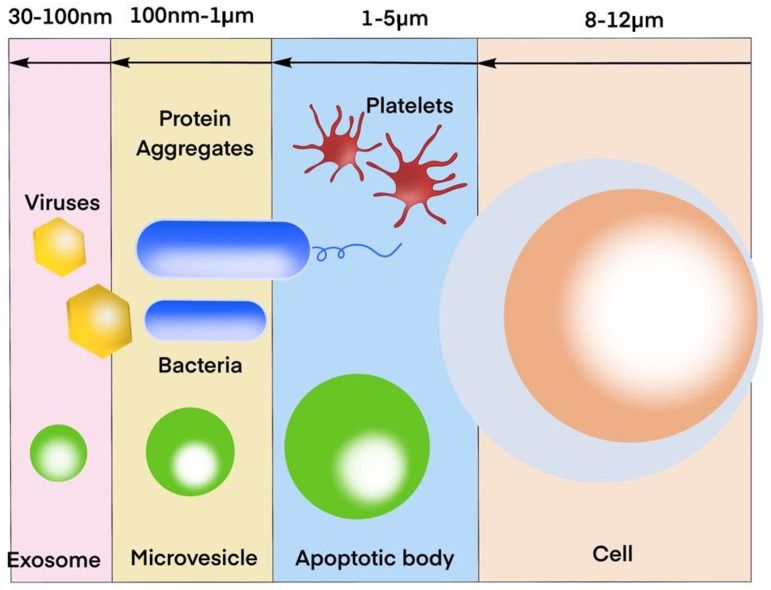
This Figure illustrates the relative sizes of various biological entities. Exosomes, ranging from 30 to 100 nm, are comparable in size to viruses. Larger extracellular vesicles, known as microvesicles, fall within the 100 nm to 1 µm range. Apoptotic bodies, which are even larger, measure between 1 µm and 5 µm. For comparison, platelets are in the 1 µm to 5 µm range, while cells are significantly larger, measuring 8–12 µm. This Figure highlights the size scale from exosomes to cells, demonstrating the hierarchical structure of these biological components.

**Figure 2:**
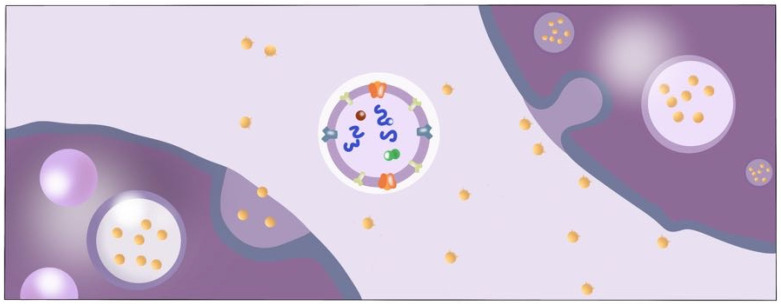
Exosomes between the cells (purple-colored) function as intercellular communicators, facilitating the transfer of proteins (green), lipids (red), and RNA (blue).

Exosomes, small membrane-bound vesicles secreted by cells, have garnered significant attention in recent years due to their potential therapeutic applications. These tiny particles, measuring between 30 and 100 nanometers in diameter, are derived from various cell types, including stem cells, immune cells, and cancer cells (Figure 1) [1,2,3]. Initially discovered as a means of cellular waste disposal, exosomes have been found to play a crucial role in intercellular communication, facilitating the transfer of proteins, lipids, and RNA between cells [3,4] (Figure 2).

The clinical applications of exosomes are vast and multifaceted. One of the most promising areas of research is in the field of regenerative medicine, where exosomes derived from stem cells have been shown to promote tissue repair and regeneration. For example, exosomes have been used to treat cardiac diseases, such as myocardial infarction, by promoting angiogenesis and improving cardiac function. Similarly, exosomes have been explored as a therapeutic agent for the treatment of neurological disorders, including Parkinson’s disease and Alzheimer’s disease [5,6].

In addition to their therapeutic potential, exosomes have also been studied for their diagnostic capabilities. Exosomal biomarkers have been identified for various diseases, including cancer, allowing for the development of non-invasive diagnostic tests [7,8]. Furthermore, exosomes have been explored as a means of delivering targeted therapeutics to specific cells or tissues, circumventing the limitations of traditional delivery methods [9].

Despite the significant progress that has been made in the field of exosome research, there are still many challenges and uncertainties that need to be addressed. For example, the optimal methods for isolating and purifying exosomes are still being debated, and the stability and bioavailability of exosomal cargo remain a concern [10,11]. Furthermore, the regulatory environment surrounding the use of exosomes as a therapeutic agent is still evolving [12].

This critical review aims to provide a comprehensive overview of the current state of knowledge in the field of clinical applications of exosomes. Through a critical analysis of the existing literature, we will explore the benefits and limitations of exosomes as a therapeutic agent, discuss the challenges and uncertainties that need to be addressed, and provide recommendations for future research directions.

Keywords including “Exosome”, “Diagnostic” “Therapy”, “Therapeutic”, “Clinical Application”, and “Clinical Implication” were searched in the MEDLINE, PubMed, and Ovid databases for relevant studies published on clinical trials, diagnosis, and treatment. Some papers were further reviewed using a double-blinding approach, sample size, control usage, randomization usage, and objective endpoint measurements. All studies were classified according to the Oxford Center for evidence-based medicine evidence hierarchy.

## 2. Chemical Properties of Exosomes

Hu et al. [13] reviews the clinical applications of exosome membrane proteins. The authors discuss the importance of exosomes in disease diagnosis, prognosis, and treatment. They highlight the potential of exosome membrane proteins as biomarkers for various diseases, including cancer, cardiovascular disease, and neurodegenerative disorders. The authors also review the use of exosomes as therapeutic agents, including their ability to deliver therapeutic cargo and modulate immune responses. The paper concludes that further research is needed to fully understand the potential benefits and risks of exosome membrane proteins (Level 5).

Santos and Almeida [14] provide an overview of the development of exosome-based vaccines, highlighting their potential as a novel approach to vaccine design. The authors review the history of exosome-based vaccines, from the discovery of exosomes in the 1980s to the current state of research. They discuss the advantages of exosomes as vaccine carriers, including their ability to deliver multiple antigens, modulate immune responses, and potentially induce long-term immunity. The authors also summarize ongoing clinical trials evaluating exosome-based vaccines for various diseases, including cancer, infectious diseases, and autoimmune disorders. The paper concludes that while exosome-based vaccines show promising results, further research is needed to fully elucidate their potential and address challenges associated with their development and manufacturing (Level 3b).

Song et al. [15] provides an overview of the emerging role of exosomes as novel therapeutics. The authors discuss the biology of exosomes, including their composition, formation, and function, as well as their potential applications in various diseases. They also review the current technologies used to isolate, purify, and characterize exosomes, as well as the clinical applications of exosomes in cancer, regenerative medicine, and immunotherapy. The authors highlight the advantages and challenges of exosome-based therapies, including their potential for targeted delivery, low toxicity, and immune tolerance. Finally, they discuss the future directions and next steps for exosome research and development (Level 5).

Aheget et al. [16] reviews the potential of exosomes as a new player in translational nanomedicine. The authors discuss the unique characteristics of exosomes, including their natural origin, biocompatibility, and ability to deliver therapeutic agents. They highlight the advantages of exosomes over traditional nanoparticles, including their ability to evade the immune system and target specific cells. The authors review the current applications of exosomes in nanomedicine, including their use as carriers for drugs, genes, and siRNAs. They also discuss the challenges and limitations associated with the use of exosomes, including their isolation and purification methods and their potential for immune rejection (Level 5).

Wang et al. [17] provides a comprehensive review of the recent progress in exosome research, focusing on their isolation, characterization, and clinical applications. The authors discuss the current methods for isolating and characterizing exosomes, including the use of novel biomarkers and techniques. They also highlight the clinical applications of exosomes, including their potential as diagnostic and therapeutic biomarkers for cancer and other diseases. The authors also explore the challenges and limitations of exosome research, including the need for standardized methods and the potential for contamination (Level 5).

Hood and Wickline [18] provide a systematic approach to the development of exosome-based translational nanomedicine. The authors highlight the potential of exosomes as a delivery platform for therapeutic agents, including siRNAs, miRNAs, and proteins. They discuss the current challenges and limitations in the development of exosome-based therapies, including the need for standardized methods for isolating and characterizing exosomes, as well as the potential for immune recognition and clearance. The authors propose a systematic approach to exosome-based translational nanomedicine, which involves the development of a comprehensive framework for designing, testing, and implementing exosome-based therapies (Level 5).

Donoso-Quezada et al. [19] provides a comprehensive review of the current state-of-the-art exosome loading and functionalization techniques for enhanced therapeutics. The authors discuss the various methods used to load and functionalize exosomes with therapeutic molecules, such as siRNAs, miRNAs, and proteins. They highlight the importance of understanding the molecular mechanisms of exosome-mediated delivery and the potential challenges and limitations of exosome-based therapies. The authors also review the recent advancements in exosome loading and functionalization techniques, including the use of nanoparticles, liposomes, and biodegradable polymers (Level 2c).

Das et al. [20] explores the potential of exosomes as a novel delivery platform for therapeutics across biological barriers. The authors discuss the unique features of exosomes, including their ability to cross cell membranes, evade immune recognition, and target specific cells. They review the current state of exosome-based delivery systems, including their use in cancer therapy, regenerative medicine, and infectious disease treatment. The authors also highlight the potential challenges and limitations of exosome-based therapies, including the need for standardization and scalability of exosome production (Level 3b).

Gurunathan et al. [21] provides a comprehensive review of the factors influencing exosome biogenesis, functions, and therapeutic applications. The authors discuss the role of various biological and environmental factors, including cell type, cell culture conditions, and disease state, in shaping exosome composition and function. They also review the current understanding of exosome-mediated cellular communication, including the transfer of proteins, lipids, and RNA molecules between cells. The authors highlight the potential therapeutic applications of exosomes, including their use in cancer treatment, regenerative medicine, and gene therapy. Additionally, they discuss the challenges and limitations associated with exosome-based therapies, including exosome isolation and purification, and the need for further research to fully understand their mechanisms of action (Level 3b).

Zhang et al. [22] provides a comprehensive review of exosomes, including their classification, isolation techniques, storage, and applications in diagnosis and targeted therapy. The authors discuss the different types of exosomes, including extracellular vesicles, microvesicles, and apoptotic bodies, and highlight the importance of standardization in exosome isolation and characterization. They also review various methods for isolating exosomes, including ultracentrifugation, density gradient centrifugation, and immune-affinity chromatography. The authors discuss the potential of exosomes as diagnostic biomarkers for various diseases, as well as their use in targeted therapy for cancer and other diseases. They also highlight the challenges associated with exosome-based therapies, including the need for improved understanding of exosome biology and the development of standardized manufacturing processes (Level 5).

Kang et al. [23] explores the potential of exosomes as theragnostics in various clinical situations. The author discusses the role of exosomes in diagnostics, particularly in the detection of biomarkers for cancer and other diseases. They also highlight the potential of exosomes as therapeutic agents, including their use in targeted therapy for cancer and other diseases. The author reviews the current state of knowledge on exosome-based therapies, including their advantages and limitations, and discusses the challenges associated with their development and translation to clinical practice. Additionally, they highlight the need for further research on the safety and efficacy of exosome-based therapies (Level 5).

Table 1 provides a summary of the chemical properties of exosomes.

## 3. Extraction and Clinical Uses of Exosomes

Mendt, Rezvani, and Shpall [24] discuss the potential use of mesenchymal stem cell-derived exosomes (MSC-Exos) as a therapeutic agent for various diseases. The authors highlight the benefits of MSC-Exos, including their ability to promote tissue repair and regeneration, modulate immune responses, and deliver therapeutic cargo. They also discuss the challenges and uncertainties associated with the use of MSC-Exos, including the need for standardized methods for isolating and purifying exosomes, as well as the potential risks associated with their use. The authors conclude that further research is needed to fully understand the potential benefits and risks of MSC-Exos and to optimize their use as a therapeutic agent (Level 4).

Perocheau et al. [25] discusses the current state of clinical applications for exosomes, highlighting their potential as therapeutic agents for various diseases. The authors review the existing evidence for exosomes in treating cardiovascular disease, cancer, and neurological disorders. They also discuss the challenges and limitations associated with the use of exosomes, including their isolation, purification, and characterization. The authors conclude that while significant progress has been made in understanding the biology of exosomes, further research is needed to fully elucidate their clinical potential (Level 3a).

The paper by Rezaie et al. [26] provides a comprehensive review of the current state of exosome-based therapies in clinical trials, highlighting their potential and challenges. The authors discuss the various ways exosomes are being used in clinical trials, including their application as therapeutic agents for cancer, autoimmune disorders, and infectious diseases. They also examine the challenges and limitations associated with exosome-based therapies, including their isolation, purification, and characterization. The authors emphasize the need for further research to address these challenges and improve the efficacy and safety of exosome-based therapies (Level 5).

Urbanelli et al. [27] reviews the potential of exosomes as a novel approach for diagnosis and therapy. The authors discuss the role of exosomes in various biological processes, including cellular communication and immune response. They highlight the advantages of exosomes as diagnostic tools, including their ability to detect specific biomarkers and provide real-time information on disease progression. The authors also explore the therapeutic potential of exosomes, including their use as vehicles for targeted drug delivery and gene therapy. They discuss the challenges and limitations associated with exosome-based strategies, including their isolation, purification, and characterization (Level 5).

Cully [28] highlights the progress of exosome-based therapies in moving into clinical trials. The author notes that several exosome-based candidates have entered clinical trials, with a focus on treating cancer, autoimmune diseases, and neurological disorders. The author also discusses the advantages of exosomes as therapeutic agents, including their ability to deliver specific biomarkers and modulate immune responses. Additionally, the author mentions the challenges and limitations associated with exosome-based therapies, including issues with scalability, purity, and stability (Level 1b).

Batrakova and Kim [29] focus on the development and regulation of exosome-based therapy products. The authors discuss the current understanding of exosomes and their potential as therapeutic agents, highlighting their ability to deliver biomarkers, modulate immune responses, and treat various diseases. The authors also provide an overview of the regulatory framework for exosome-based therapies, including the challenges and limitations associated with their development and approval. They discuss the need for the standardization and validation of exosome-based products, as well as the importance of understanding the biological mechanisms underlying exosome-mediated delivery (Level 2b).

Harrell et al. [30] provides an overview of the therapeutic potential of exosomes derived from mesenchymal stem cells (MSCs). The authors discuss the current understanding of exosomes, their biology, and their ability to deliver therapeutic cargo to target cells. They highlight the benefits of using MSC-derived exosomes, including their immunomodulatory and anti-inflammatory properties, as well as their potential to treat various diseases such as cardiovascular disease, neurological disorders, and cancer. The authors also summarize the current state of clinical trials and regulatory frameworks for exosome-based therapies, emphasizing the need for further research to address the challenges and limitations associated with their translation into clinical practice (Level 5).

The paper by Lee et al. [31] provides an overview of the therapeutic features and clinical trials of mesenchymal stem cell (MSC)-derived exosomes. The authors discuss the composition and functions of MSC-derived exosomes, highlighting their potential for therapeutic applications in various diseases, including cardiovascular, neurological, and inflammatory disorders. They review the current state of MSC-derived exosome-based therapies in preclinical studies and clinical trials, focusing on their mechanisms of action, safety, and efficacy. The authors also highlight the challenges and limitations of MSC-derived exosome-based therapies, emphasizing the need for further research to fully understand their potential and address these limitations (Level 1c).

Chen et al. [32] reviews the current state of exosomes in clinical trials, focusing on their production and compliance with good manufacturing practice (GMP). The authors discuss the challenges and limitations of exosome production, including the need for standardized methods and quality control. They highlight the importance of GMP-compliant production to ensure the safety and efficacy of exosome-based therapies. The review also discusses the current regulatory landscape and the potential for exosomes to be used as a new therapeutic platform. The authors conclude that while there are still challenges to be overcome, the development of GMP-compliant exosome production methods holds promise for the future of exosome-based therapies (Level 3b).

Lotfy et al. [33] reviews the current status of clinical trials involving mesenchymal stromal/stem cell (MSC)-derived exosomes. The authors summarize the results of 14 clinical trials which investigated the use of MSC-derived exosomes as a treatment for various diseases, including cardiovascular disease, graft-versus-host disease, and inflammatory bowel disease. The authors highlight the potential benefits of exosomes, including their ability to promote tissue repair and modulate the immune response. They also discuss the challenges and limitations of exosome therapy, including the need for the standardization of exosome production and purification methods. The authors conclude that MSC-derived exosomes show promise as a therapeutic agent, but further research is needed to fully understand their efficacy and potential (Level 2b).

Table 2 provides a summary of the extraction and clinical uses of exosomes.

## 4. Exosomes in Disease Treatment and Regenerative Medicine

Chung et al. [34] provide an overview of the current uses and future applications of exosomes. The authors discuss the role of exosomes in disease diagnosis, prognosis, and treatment, highlighting their potential as biomarkers for various diseases. They also review the use of exosomes as therapeutic agents, including their ability to deliver therapeutic cargo and modulate immune responses. The authors explore the potential applications of exosomes in regenerative medicine, cancer therapy, and gene therapy. Additionally, they discuss the challenges and limitations associated with the use of exosomes, including their isolation, purification, and characterization (Level 5).

Vitha et al. [35] explore the potential of exosomes in orthopedics. The authors provide an overview of exosomes, their biogenesis, and their characteristics. They highlight the therapeutic applications of exosomes in orthopedic disorders, including their ability to deliver bioactive molecules, modulate immune responses, and promote tissue repair. The authors discuss the potential uses of exosomes in treating conditions such as osteoarthritis, osteoporosis, and tendon injuries. They also review the current state of exosome-based therapies in preclinical studies and clinical trials, emphasizing the need for further research to fully understand their mechanisms and potential (Level 2c).

Popowski et al. [36] discuss the potential of exosomes in lung regenerative medicine. The authors provide an overview of exosomes, their biogenesis, and their therapeutic applications. They highlight the benefits of using exosomes, including their ability to deliver therapeutic cargo, modulate immune responses, and promote tissue repair. The authors focus on the use of exosomes for treating lung diseases such as chronic obstructive pulmonary disease (COPD), pulmonary fibrosis, and lung cancer. They review the current state of exosome-based therapies in preclinical studies and clinical trials, emphasizing the need for further research to fully understand their mechanisms (Level 2b).

Sanghani et al. [37] review the current state of exosome therapies in ophthalmology, highlighting their potential applications in treating various eye diseases. The authors discuss the basic science and preclinical studies that have demonstrated the therapeutic potential of exosomes in ophthalmology, including their ability to deliver therapeutic cargo, modulate immune responses, and promote tissue repair. They also review the current clinical trials and case reports that have used exosomes to treat various ophthalmic disorders, such as age-related macular degeneration, diabetic retinopathy, and glaucoma. The authors emphasize the need for further research to fully understand the mechanisms of action and efficacy of exosome therapies in ophthalmology (Level 2c).

Kost et al. [38] provide a comprehensive review of the current state of exosome therapy in hair regeneration. The authors summarize the existing literature on the use of exosomes as a potential treatment for hair loss, including their potential mechanisms of action, such as stimulating hair growth and improving hair follicle function. They highlight the benefits of exosomes, including their ability to target specific cells and tissues, and their potential for reducing side effects. The authors also discuss the challenges and limitations of exosome therapy, including the need for standardization of exosome production and purification methods. They conclude that while exosome therapy shows promise for hair regeneration, further research is needed to fully understand its efficacy and potential (Level 2c).

Kavya et al. [39] provide a comprehensive review of the therapeutic applications of exosomes in various diseases. The authors summarize the existing literature on the use of exosomes as a potential treatment for various diseases, including cancer, cardiovascular disease, neurological disorders, and infectious diseases. They highlight the potential benefits of exosomes, including their ability to deliver therapeutic molecules, modulate the immune response, and promote tissue repair. The authors also discuss the challenges and limitations of exosome therapy, including the need for standardization of exosome production and purification methods. They conclude that exosomes have great potential for treating various diseases, but further research is needed to fully understand their efficacy and potential (Level 1c).

Salarpour et al. [40] reviews the potential of exosomes and exosome-nanoparticle complexes for treating brain disorders. The authors discuss the unique features of exosomes, including their ability to cross the blood–brain barrier and deliver therapeutic agents to specific brain regions. They highlight the potential benefits of using exosomes as a targeted delivery system for brain disorders, including Alzheimer’s disease, Parkinson’s disease, and stroke. The authors also review the current state of research on exosome–nanoparticle complexes, which combine the targeting capabilities of exosomes with the enhanced delivery efficiency of nanoparticles. They conclude that further research is needed to fully understand the potential of exosomes and exosome–nanoparticle complexes for treating brain disorders (Level 1b).

Table 3 provides a summary of the exosomes in disease treatment and regenerative medicine.

**Figure 3:**
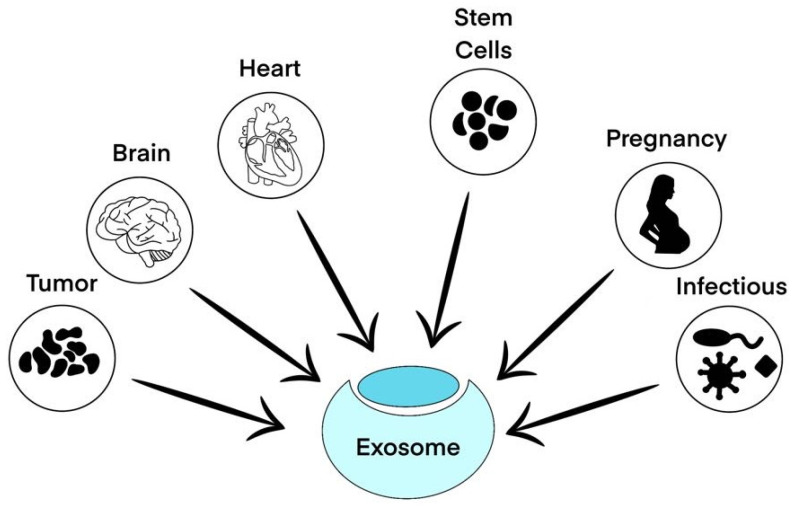
The diverse medical applications of exosomes. The arrows represent the various sources and applications of exosomes in different medical fields. The versatile roles of exosomes in advancing medical science offer novel approaches for diagnosis, treatment, and monitoring of various health conditions.

The exosomes can be used in many of the following medical fields (Figure 3).

## 5. Exosomes in Diagnostic and Therapeutic Tools

Huda et al. [42] provide a comprehensive review of the potential uses of exosomes in biomedical applications, including diagnosis and targeted drug delivery. The authors discuss the current state of exosome-based diagnostics, highlighting their ability to detect biomarkers for various diseases, such as cancer, neurological disorders, and infectious diseases. They also explore the therapeutic potential of exosomes, including their use as vehicles for targeted drug delivery and gene therapy. The authors summarize the progress in clinical and preclinical applications, highlighting the challenges and limitations associated with exosome-based therapies (Level 2b).

Sun and Liu [43] review the potential of cancer cell-derived exosomes in clinical applications. The authors discuss the role of exosomes in cancer development and progression, as well as their ability to mediate immune evasion and promote tumor growth. They also highlight the recent advances in understanding the biological properties of exosomes, including their composition, isolation, and characterization. The authors review the potential therapeutic applications of cancer cell-derived exosomes, including their use as biomarkers for cancer diagnosis and treatment, as well as their potential for targeted delivery of therapeutic agents. They also discuss the challenges and limitations associated with the use of exosomes in clinical applications, including their heterogeneity and potential for immune rejection (Level 2b).

Xu et al. [44] review the potential of exosome-based immunotherapy for cancer treatment. The authors discuss the biological properties of exosomes, including their ability to deliver therapeutic agents, such as siRNAs, miRNAs, and proteins, to specific cells. They highlight the advantages of exosome-based immunotherapy, including its potential for targeted delivery, low toxicity, and immune tolerance. The authors review the current preclinical and clinical studies using exosome-based immunotherapy for cancer treatment, including its use in combination with other therapies such as chemotherapy and immunotherapy. They also discuss the challenges and limitations associated with the use of exosome-based immunotherapy, including its potential for immune rejection and the need for improved manufacturing and characterization techniques (Level 2c)

Zipkin and colleagues [45] provide a comprehensive review of exosomes, their biology, and their potential applications in medicine. The author highlights the recent advancements in exosome research, including their isolation and purification methods and their potential as therapeutic agents. The review also discusses the challenges and limitations associated with exosome research, including the lack of standardization and the need for further understanding of their biology and mechanisms of action. The author also discusses the potential applications of exosomes in various fields, including cancer therapy, regenerative medicine, and vaccine development (Level 5).

Tai et al. [46] reviews the current understanding of exosomes in cancer development and their potential applications in clinical settings. The authors discuss the role of exosomes in cancer progression, including their involvement in tumor growth, metastasis, and immune evasion. They also highlight the potential of exosomes as diagnostic biomarkers, therapeutic agents, and vaccine delivery vehicles. The review covers the current state of exosome-based therapies, including their advantages and limitations, and discusses the challenges and opportunities in translating exosome research into clinical practice. The authors conclude that exosomes have great potential for improving cancer diagnosis and treatment, but further research is needed to fully understand their mechanisms and optimize their therapeutic applications (Level 5).

Skuratovskaia et al. [47] review the current state of exosomes in the treatment of inflammatory diseases. The authors highlight the potential benefits of exosomes as a therapeutic platform, including their ability to deliver therapeutic molecules and modulate the immune response. However, they also discuss the limitations of exosome-based therapies, including issues with scalability, standardization, and stability. The review covers the challenges of exosome production, purification, and characterization, as well as the difficulties in delivering exosomes to target tissues and organs. The authors conclude that while exosomes show promise for treating inflammatory diseases, further research is needed to overcome these limitations and optimize their therapeutic potential (Level 4).

Lu et al. [48] investigate the potential of exosome-derived biomarkers for diagnosing and monitoring atherosclerosis. The authors analyzed exosomal RNA and protein cargo from the plasma samples of patients with atherosclerosis and healthy controls. They found that exosomal miR-126-3p and PD-L1 were significantly increased in patients with atherosclerosis, while exosomal miR-145-5p was decreased. The authors used these biomarkers to develop a diagnostic model that showed high accuracy in distinguishing patients with atherosclerosis from healthy controls. The study also explored the clinical application of exosome-derived biomarkers in patients with coronary artery disease (Level 1b).

Dimik et al. [49] provide a comprehensive review of the current therapeutic applications and potential capabilities of exosomes in human reproduction. The authors discuss the role of exosomes in fertility, pregnancy, and reproductive health, including their ability to deliver therapeutic molecules and modulate the immune response. They also highlight the potential uses of exosomes in assisted reproductive technologies, such as in vitro fertilization and embryo transfer. The review covers the current state of research on exosomes in human reproduction, including their mechanism of action, advantages, and challenges. The authors conclude that exosomes have great potential for improving human reproductive health and fertility outcomes (Level 2c).

He et al. [50] provide an overview of the concept of exosome theranostics, which involves the use of exosomes as both therapeutic agents and diagnostic tools. The authors discuss the biology of exosomes, including their origin, composition, and function, as well as their potential applications in various diseases. They highlight the advantages of exosomes as a delivery platform, including their ability to target specific cells and tissues and their potential for reducing side effects. The authors also review the current state of exosome-based theranostics in cancer, cardiovascular disease, and neurological disorders. They conclude that exosome theranostics has great potential for improving disease diagnosis and treatment (Level 5).

Dorayappan et al. [51] discuss the role of exosomes in ovarian cancer, focusing on their biological significance and potential clinical applications. The authors review the current literature on exosome biology, including their origin, composition, and function in ovarian cancer. They highlight the potential of exosomes as diagnostic and therapeutic tools, including their ability to deliver therapeutic molecules and modulate the immune response. The authors also discuss the potential uses of exosomes in ovarian cancer diagnosis, including their ability to detect specific biomarkers and monitor disease progression. They conclude that exosomes have great potential for improving our understanding of ovarian cancer and developing new treatments (Level 1b).

Lorenc et al. [52] provide an overview of the current perspectives on the clinical use of exosomes as a personalized contrast to media and theranostics. The authors discuss the potential applications of exosomes in various medical fields, including imaging, diagnosis, and therapy. They highlight the advantages of exosomes, such as their ability to target specific cells and tissues and their potential for personalized medicine. The authors also discuss the challenges and limitations of exosome research, including the need for the standardization of exosome production and purification methods. They conclude that exosomes have great potential for clinical use, but further research is needed to fully understand their properties and potential (Level 5).

Codispoti et al. [53] introduces a novel approach to regenerative medicine using NANOmetric BIO-banked MSC-derived exosomes (NANOBIOME). The authors describe the development of a proprietary method for isolating and preserving exosomes derived from mesenchymal stem cells (MSCs). The NANOBIOME technology involves the use of nanotechnology to enhance the stability and potency of the exosomes, which are then banked for future use. The authors highlight the potential advantages of this approach, including the ability to standardize and scale up exosome production and the potential for use in a wide range of therapeutic applications. The authors also discuss the initial results of preclinical studies using NANOBIOME, which show promising results in terms of tissue repair and regeneration (Level 2c).

Tang et al. [54] provide a comprehensive review of the clinical implications, applications, and challenges of cancer exosomes. The authors discuss the role of exosomes in cancer progression, metastasis, and immune evasion, and highlight their potential as diagnostic and therapeutic biomarkers. They also explore the challenges and limitations of exosome-based research, including the need for standardization of exosome isolation and characterization methods. The authors also discuss the potential applications of exosomes in cancer therapy, including the use of exosomes as vectors for cancer gene therapy, and the development of exosome-based immunotherapy. The authors conclude that while there are many challenges to overcome, cancer exosomes have great potential for improving cancer diagnosis and treatment (Level 5).

Willis et al. [41] discuss the potential of exosomes as a therapeutic platform for cardiovascular diseases. The authors highlight the challenges in developing exosome-based therapies, including the need for standardized methods for isolating and characterizing exosomes. They discuss the heterogeneity of exosomes and the importance of understanding their biological functions and potency. The authors also explore the concept of “fit-for-purpose” potency, which refers to the ability of exosomes to achieve specific therapeutic goals. They conclude that further research is needed to develop exosome-based therapies that can effectively treat cardiovascular diseases (Level 2c).

Wang et al. [55] explore the potential of exosomes as a novel therapeutic approach for targeting cancer stem cells. The authors discuss the unique features of exosomes, including their ability to selectively target cancer cells and evade immune recognition. They review the current understanding of exosome-based cancer therapy, including the use of exosomes to deliver therapeutic agents, such as siRNAs and chemotherapeutics, to cancer cells. The authors highlight the potential benefits of exosome-based therapy, including improved targeting of cancer stem cells and reduced side effects compared to traditional chemotherapy. They also discuss the challenges and limitations associated with exosome-based therapies, including the need for standardization and scalability of exosome production (Level 5).

Tzng et al. [56] highlight the current challenges and limitations surrounding the development of exosome-based treatments. The authors discuss the lack of standardization and scalability in exosome production, which hinders the translation of exosome-based therapies from bench to bedside. They also emphasize the need for improved characterization and tracking of exosomes, as well as the development of robust manufacturing processes to ensure consistency and quality. The authors also highlight the challenges associated with regulatory approval and reimbursement, as well as the need for further research on the safety and efficacy of exosome-based therapies. They conclude that addressing these challenges will be crucial for the successful development of exosome-based treatments (Level 2b).

Nafar et al. [57] review the potential of exosomes as a target for cancer treatment. The authors discuss the role of exosomes in cancer development and progression, and highlight their potential as therapeutic agents for cancer treatment. They review the current state of knowledge on exosome-based therapies, including their use in targeted therapy, immunotherapy, and gene therapy. The authors also discuss the challenges associated with exosome-based therapies, including the need for improved understanding of exosome biology and the development of standardized manufacturing processes (Level 2b).

Table 4 provides a summary of the exosomes in diagnostic and therapeutic tools.

## 6. Recent Derivatives from Humans and Plants

Exosomes serve as vital messengers within the dermis, significantly influencing the behavior of fibroblasts, the cells primarily responsible for producing collagen and elastin. These proteins are essential for maintaining skin elasticity and strength. Exosomes facilitate communication between skin cells and fibroblasts, enhancing collagen and elastin synthesis, and increasing dermal fat, thereby promoting the skin’s regenerative and restorative capacities for anti-aging. This leads to improved skin texture and a reduction in wrinkles and fine lines. Additionally, exosomes contribute to elastin production, which is crucial for maintaining a youthful and firm skin appearance [58].

At the molecular level, exosomes exert their rejuvenating effects through various pathways and growth factors, notably TGF-B. TGF-B plays a critical role in skin repair and rejuvenation by influencing cell growth, proliferation, and differentiation. Exosomes carry and deliver TGF-B to target cells in the skin, triggering specific signaling cascades that improve skin structure and function.

Furthermore, exosomes are involved in the modulation of the extracellular matrix (ECM), a complex network of proteins and other molecules that provide structural and biochemical support to surrounding cells. They assist in remodeling this matrix, which is particularly important in wound healing and the prevention of scar formation.

**Figure 4:**
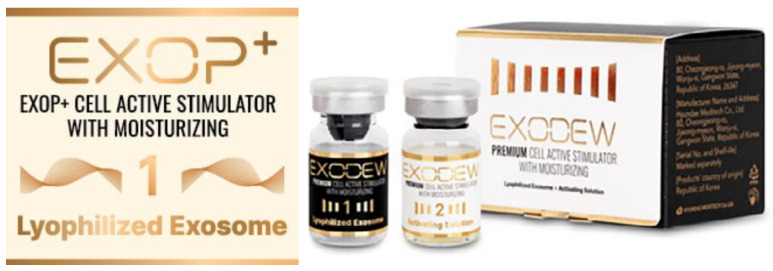
There has been a recent surge in interest in exosomes derived from the human pharynx (EXOP by Sihler Inc., Seoul, Republic of Korea, and Exodew by Hyundai Meditech Inc., Wonju, Republic of Korea). Stem cells collected via swab-based sampling during pharyngeal examinations, such as those conducted for early childhood influenza, are recognized for their outstanding differentiation abilities.

**Figure 5:**
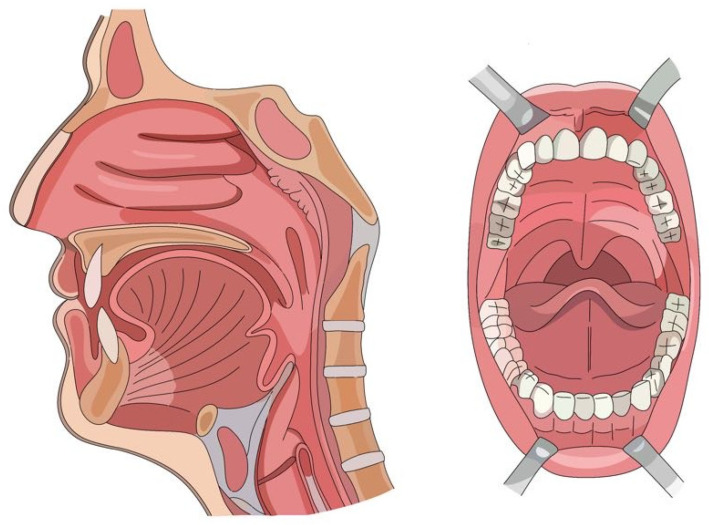
Pharyngeal stem cells obtained through swab-based sampling during examinations, such as influenza screenings in early childhood, are recognized for their remarkable differentiation capabilities.

Recently, there has been a growing focus on exosomes derived from the human pharynx (EXOP, Sihler Inc., Seoul, Republic of Korea and Exodew, Hyundaimeditech Inc., Seoul, Republic of Korea) (Figure 4). Stem cells collected through swab-based sampling during examinations, such as influenza screenings in early childhood, are known for their exceptional differentiation capabilities (Figure 5) [59].

In the realm of plant-based research, ginseng-derived exosomes have been actively explored, leveraging the long-established benefits of ginseng in Asian countries, where it has been extensively studied and used in cosmetics. This has led to the emergence of companies dedicated to studying exosomes extracted from ginseng (Exodew, Hyundaimeditech Co., Wonjusi, Republic of Korea) being a notable product. Ginseng, renowned for its anti-inflammatory, anti-cancer, immunostimulant, and osteogenic/anti-osteoporotic properties, has recently been utilized to derive exosomes.

A study by Seo et al. [60] confirmed that these exosomes inhibit osteoclast differentiation, and investigated the underlying molecular mechanisms.

Exosomes were isolated using centrifugation with a sucrose gradient, and their properties were analyzed through dynamic light scattering, zeta potential measurements, and transmission electron microscopy. Bone marrow-derived macrophages (BMMs) were used to assess the cytotoxicity of exosomes and their ability to inhibit osteoclast differentiation. The results demonstrated that exosomes maintained high BMM viability and proliferation while inhibiting osteoclastogenesis. At concentrations greater than 1 μg/mL, the exosomes significantly impeded osteoclast differentiation, as confirmed by tartrate-resistant acid phosphatase and F-actin staining.

Exosomes also suppressed the RANKL-induced signaling pathways, including IκBα, c-JUN N-terminal kinase, and extracellular signal-regulated kinase, as well as the genes regulating osteoclast maturation. The exosomes were rich in Rb1 and Rg1 ginsenosides, and were more effective at inhibiting osteoclast differentiation than these ginsenosides alone or in combination.

Additionally, the unique properties of exosomes can be applied in esthetics. Their anti-inflammatory and immunostimulant effects could be beneficial for skin health, potentially aiding in the treatment of inflammatory skin conditions and enhancing skin rejuvenation. The osteogenic properties might also support facial bone health, contributing to a more youthful appearance.

Another study by Jang et al. [61] highlighted that exosomes are nano-sized extracellular vesicles that play a crucial role in regulating cell growth and defense by delivering bioactive cellular components. They hold significant promise for biomedical and cosmetic applications, particularly from medicinal crops like ginseng. However, isolating stable exosomes with high purity remains a major challenge.

This study tested three methods to isolate exosomes from ginseng: ultracentrifugation (the most widely used method), the ExoQuick system (a polymer-based exosome precipitation approach), and a combination of both methods. Size distribution analysis showed that the purity of exosome isolation was 34.1% with ultracentrifugation, 59.7% with ExoQuick, and significantly improved to 83.3% with the combination method.

The combination method not only enhanced exosome isolation purity, but also increased the colloidal stability of the isolated ginseng exosomes, almost doubling the stability achieved by ultracentrifugation alone. Additionally, this method was effective in isolating high-purity and high-stability exosomes from the model plant Arabidopsis.

Overall, these findings indicate that the combination method is highly suitable for isolating high-purity and high-stability exosomes from plants, including ginseng, making it a promising approach for both biomedical and cosmetic uses.

## 7. Emphasizing the Importance of the Donor Source for Exosomes

**Figure 6:**
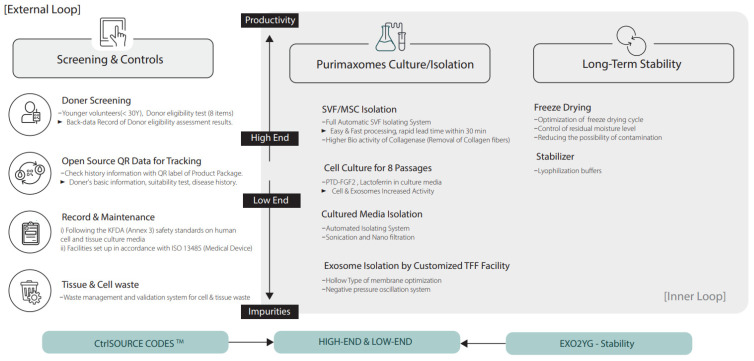
Recent technology using screening to keep the health data of donors and inspections of exosomes.

When producing exosomes for therapeutic purposes, variations depending on the donor must be considered because various donors can produce exosomes with diverse forms of functional characteristics. The recent literature underscores the critical importance of the exosome source in determining quality, safety, and potency, with donor age particularly influencing regulatory capacity, proliferative potential, and differentiation capacity. Furthermore, a vigilant review on the harvesting and selection procedures of donor cells, donor suitability criteria, health status, and medical history shall be made. ZISHEL BIO, a leading exosomal biotech company based in South Korea, has set a pioneering standard to seamlessly integrate an in-house standard for screening and harvesting donor cells in partnership with is in-house certified lab clinic. Stressing the importance of donor source cells, the company leads comprehensive donor profiling, virus checking, and harvesting services, following rigorous, standardized methods. General steps from screening, keeping health data, and inspections are as follows (Figure 6).

It is essential to screen for signs of infection before collecting tissue and cells from a donor. The ‘Cell Therapy Donor Suitability Evaluation Guidelines’ apply to the suitability criteria for donors who provide cells or tissues for exosome production.

(1) Procedure for Doner’s Screening and Testing for Stem Cell Therapy

* Doner conducts the below procedures in accordance with the international guidelines and national regulations.

(*) Donor Test Standard Parameters

According to the Human Cell Tissue Culture Safety Standards, donors are checked for the following infectious diseases:

(1) Hepatitis B Virus (HBV), Hepatitis C Virus (HCV), Human Immunodeficiency Virus (HIV), Human T-lymphotropic virus (HTLV), ParvovirusB19, Cytomegalovirus (CMV), and Epstein–Barr virus (EBV) Infectious Diseases.
(2) Transmissible spongiform encephalopathy and suspicion on transmissible spongiform encephalopathy.
(3) Infections caused by bacteria such as chlamydia, gonorrhea, and tuberculosis Syphilis Treponema.
(4) Sepsis and suspected sepsis.
(5) Congenital or chronic diseases that can affect cellular structure.

(2) Open-Source QR Data of the Donor Cells

Doner’s basic information, suitability test, disease history, etc., can be provided using a QR code. By providing this information about the donor, we provide trust and stability to patients receiving exosome treatment.

** QR Data of the Doner: Gender, Age, Harvested cell type, Date/Site of Collection, Donor Eligibility Assessment Results

Again, exosomes have emerged as a therapeutic option for regenerative medicine and promoting overall health. As their potential as therapeutics is attracting attention, many exosome biotech companies are making efforts to develop and produce therapeutic exosomes. To develop and commercialize the exosome therapeutics, they must develop a new production scheme with the added standardization and validation of exosomes. Scalability, purity, and stability considerations shall be taken into account from the initial source cell harvesting step to the production and isolation step. Such considerations in terms of production and quality are detailed as follows.

(1) Overall Production

Since source cells from the donor are used to produce potent and high-purity-based exosomes, the characteristics profile of stem cells used as a starting source must be analyzed and recorded. In addition to the donor source suitability evaluation, the following analyses of the stem cells are made to ensure identification and genetic stability of the source cells. In addition, standardized methods to evaluate the potency and reproducibility of the exosomes based on various criteria are followed to ensure consistent exosome production with added quality and consistency.

* Identification of cells (chromosomal karyotype analysis, cell surface phenotypic markers, specific gene expression levels, etc.).
* Stability (shape, growth characteristics, etc.), genetic stability (karyotype analysis, etc.), microbial test (virus, bacteria, fungus, mycoplasma, etc.).
* Proof of standardization and reproducibility of the exosome production process (density, survival rate, cell properties, subculture method, culturing time carbon dioxide concentration, culture temperature, medium additives, medium composition, culture vessel, etc.)
* Standardization for isolating and purifying exosome (TFF-exosome isolation).

Every step, from sourcing to the isolation of exosomes, must adhere to the highest production standards to guarantee optimal efficacy. As highlighted by Théry et al. [62], factors such as processing and storage significantly impact exosome physiology and, consequently, influence exosome research. ZISHEL BIO’s distinctive all-in-one production pipeline prioritize efficiency and purity. Notably, the harvesting-to-culturing gap time is minimized to less than 5 min. Such practice allows our team to avoid long time delivery/storage and freezing and thawing process of the harvested media, minimizing every possible risk of disturbing the cells. This streamlined approach results in a substantial increase in both cell and exosome potency and viability. Importantly, this rapid processing occurs without introducing any mutations or stress factors that could compromise the safety and efficacy of the final product.

(2) Quality Considerations for the Optimization of Produced Exosomes

After producing exosomes, standardized guidelines for quality control shall be formulated in every possible process.

* Various characteristics of exosomes produced through the separation process must be analyzed (analyzing the profiles of exosome nano particles, size, protein, mRNA, and lipid).
* Qualitative/quantitative testing methods and acceptable limits for impurities must be established.
* Mycoplasma, viruses, bacteria, fungi, endotoxin.
* The quality and stability of extracellular vesicle treatments are tested according to the ‘Stability Test Standards for Pharmaceuticals’, etc. (FDA guide).

Although there are still many challenges and uncertainties that need to be addressed, the recent development of standardized methods for isolating and purifying exosomes is led by ZISHEL BIO, a leading biotech company for exosomes R&D based in South Korea. Powered by ZISHEL BIO’s puriMAX Technology, a customized solution for maximizing purity and standardized exosomes, purimaxomes are controlled in-house through validations from screening of harvested cells to culturing and isolation. The MSC-derived cultivated cells are screened and quality controlled via its proprietary process known as ctrlSource codes. The health data and inspections are recorded and made traceable. Purimaxomes’ culture and isolation processes are developed and classified under high- and low-end production technologies. To increase the overall production capability and consistency of exosome production, puriMax Technology is built as the innovative double-looped scheme to tackle some of the unmet challenges.

## 8. Future Insights and Potential Plans

This review of the literature highlights the vast potential of exosomes as therapeutic agents in various diseases, including cancer, cardiovascular disease, neurological disorders, and autoimmune disorders. The benefits of exosomes as therapeutic agents are numerous, including their ability to deliver therapeutic cargo, modulate immune responses, and promote tissue repair. The results of preclinical studies and clinical trials demonstrate the efficacy and safety of exosome-based therapies, which have shown promise in treating various diseases.

However, there are also several limitations and challenges associated with exosome-based therapies. One of the main challenges is the standardization and validation of exosome-based products, including issues with scalability, purity, and stability. Additionally, the regulatory framework for exosome-based therapies is still evolving, and there is a need for further research to understand the biological mechanisms underlying exosome-mediated delivery [20].

Despite these challenges, there are several promising areas of research that hold great potential for the development of exosome-based therapies. For example, the use of mesenchymal stem cell-derived exosomes shows promise in treating various diseases, including cardiovascular disease, neurological disorders, and autoimmune disorders. The development of exosome-based vaccines is another area of research that has shown promising results in preclinical studies [14].

In addition to these areas of research, there are several other potential applications of exosomes that warrant further investigation. For example, the use of exosomes in orthopedics could potentially treat conditions such as osteoarthritis, osteoporosis, and tendon injuries [35,36]. The use of exosomes in lung-regenerative medicine could potentially treat diseases such as chronic obstructive pulmonary disease (COPD), pulmonary fibrosis, and lung cancer [36,44,54].

Despite the promising results of exosome-based therapies in various diseases, there are several challenges and limitations that need to be addressed. One of the main challenges is the standardization and validation of exosome-based products, including issues with scalability, purity, and stability. Additionally, the regulatory framework for exosome-based therapies is still evolving, and there is a need for further research to understand the biological mechanisms underlying exosome-mediated delivery.

Another challenge is the difficulty in tracking the fate of exosomes after administration. Exosomes are small particles that can be taken up by various cells and tissues, making it difficult to track their fate and determine their efficacy. Additionally, the development of methods for tracking the fate of exosomes is essential for understanding their mechanisms of action and optimizing their therapeutic potential.

Several areas of research would be beneficial in advancing our understanding of exosome-based therapies and their potential applications, such as the following:

* Further research is needed to develop methods for standardizing and validating exosome-based products.
* The regulatory framework for exosome-based therapies needs to be further developed and clarified.
* The biological mechanisms underlying exosome-mediated delivery need to be fully understood.
* The development of methods for tracking the fate of exosomes needs to be advanced.
* The use of exosomes in combination with other therapeutic agents needs to be explored.
* The development of personalized therapies using patient-derived exosomes needs to be investigated.

In addition to these areas of research, there are several other potential applications of exosomes that warrant further investigation, as follows:

* The use of exosomes in regenerative medicine could potentially treat a range of conditions, including osteoarthritis, osteoporosis, and tendon injuries.
* The use of exosomes in gene therapy could potentially treat a range of genetic disorders.
* The use of exosomes in vaccine development could potentially treat a range of diseases, including infectious diseases and cancer.
* The use of exosomes in diagnostic medicine could potentially be used as a diagnostic tool for a range of diseases.

## 9. Conclusions

The clinical applications of exosomes is a rapidly evolving field with significant potential to revolutionize the treatment of various diseases. The data presented in this review highlight the versatility and efficacy of exosomes as a therapeutic agent, with promising results in the treatment of cardiovascular disease, cancer, and neurological disorders. The ability of exosomes to deliver targeted therapeutics, evade immune recognition, and promote tissue repair and regeneration make them an attractive option for clinicians.

However, despite the significant progress that has been made, there are still many challenges and uncertainties that need to be addressed. The development of standardized methods for isolating and purifying exosomes, as well as the optimization of exosomal cargo loading and stability, are critical areas of research that require further attention. Furthermore, the regulatory environment surrounding the use of exosomes as a therapeutic agent is still evolving, and guidelines for their clinical use are needed.

Future research directions should focus on addressing these challenges and uncertainties. The development of biomarkers for exosomal cargo and the optimization of exosomal delivery methods are critical areas of research that have the potential to significantly improve the efficacy and safety of exosome-based therapies. Additionally, the exploration of exosomes as a diagnostic tool for various diseases is an area that warrants further investigation.

## Figures and Tables

**Table 1:** Key points and classification of the chemical properties of exosomes.

Authors	Focus	Key Points	Conclusion	Evidence Level
Hu et al. [13]	Clinical applications of exosome membrane proteins.	Importance in disease diagnosis, prognosis, and treatment. Potential as biomarkers.	Further research needed to understand benefits and risks.	5
Santos, Almeida [14]	Development of exosome-based vaccines.	Advantages as vaccine carriers. Ongoing clinical trials for various diseases.	Promising results, but further research needed.	3b
Song et al. [15]	Emerging role of exosomes as therapeutics.	Biology, isolation, purification, and clinical applications.	Advantages and challenges discussed.	5
Wang et al. [17]	Progress in exosome research.	Isolation, characterization, and clinical applications.	Need for standardized methods and the addressing of contamination issues.	5
Zhang et al. [22]	Exosomes in diagnosis and targeted therapy.	Classification, isolation techniques, storage, diagnostic biomarkers, and targeted therapy.	Challenges in understanding biology and in standardizing processes.	5
Kang et al. [23]	Exosomes as theragnostics.	Role in diagnostics, therapeutic agents, advantages, and limitations.	Further research needed for safety and efficacy.	5
Das et al. [20]	Exosomes as a delivery platform for therapeutics.	Unique features: crossing membranes, immune evasion, and targeting cells.	Challenges in standardization and scalability.	3b
Gurunathan et al. [21]	Exosome biogenesis, functions, and therapeutic applications.	Factors influencing biogenesis, cellular communication, and therapeutic uses.	Challenges in isolation and purification, and a need for further research.	3b
Donoso-Quezada et al. [19]	Exosome loading and functionalization techniques.	Methods for loading therapeutic molecules and recent advancements.	Importance of molecular mechanisms and challenges in therapies.	2c
Aheget et al. [16]	Exosomes in translational nanomedicine.	Unique characteristics, biocompatibility, and targeted delivery.	Challenges in isolation, purification, and potential immune rejection.	5
Hood, Wickline [18]	Exosome-based translational nanomedicine.	Delivery platform for siRNAs, miRNAs, and proteins.	Challenges in standardization, immune recognition, and clearance.	5

**Table 2:** Key points and classification of the extraction and clinical uses of exosomes.

Authors	Focus	Key Points	Conclusion	Evidence Level
Mendt, Rezvani, Shpall [24]	MSC-derived exosomes as therapeutic agents.	Benefits: tissue repair, immune modulation, and therapeutic delivery. Challenges: standardization, and risks.	Further research needed to optimize usage and to understand the risks.	4
Perocheau et al. [25]	Clinical applications of exosomes.	Potential in treating cardiovascular disease, cancer, and neurological disorders.	Further research needed to understand the clinical potential.	3a
Rezaie et al. [26]	Exosome-based therapies in clinical trials.	Applications in cancer, autoimmune disorders, and infectious diseases.	Need for further research to improve efficacy and safety.	5
Batrakova, Kim [29]	Development and regulation of exosome therapy products.	Understanding, regulation, standardization, and validation of exosome-based products.	Need for further research on the biological mechanisms.	2b
Harrell et al. [30]	Therapeutic potential of MSC-derived exosomes.	Immunomodulatory, anti-inflammatory properties, and treating various diseases.	Need for further research on clinical trials and regulatory frameworks.	5
Lee et al. [31]	MSC-derived exosome therapies.	Therapeutic applications, mechanisms, safety, and efficacy.	Challenges and limitations emphasized.	1c
Lotfy et al. [33]	MSC-derived exosomes in clinical trials.	Treating cardiovascular disease, GVHD, and inflammatory bowel disease.	Need for standardization in production and purification methods.	2b
Chen et al. [32]	Exosomes in clinical trials.	Production, compliance with GMP, and the regulatory landscape.	Promising future, with challenges to overcome.	3b
Urbanelli et al. [27]	Exosomes for diagnosis and therapy.	Advantages as diagnostic tools and therapeutic vehicles.	Challenges in isolation, purification, and characterization.	5
Cully et al. [28]	Exosome-based therapies in clinical trials.	Treating cancer, autoimmune diseases, and neurological disorders.	Advantages and challenges discussed.	1b

**Table 3:** Key points and classification of the exosomes in disease treatment and regenerative medicine.

Authors	Focus	Key Points	Conclusion	Evidence Level
Chung et al. [34]	Current uses and future applications of exosomes.	Role in diagnosis, prognosis, treatment, regenerative medicine, cancer therapy, and gene therapy.	Challenges in isolation, purification, and characterization.	5
Vitha et al. [35]	Exosomes in orthopedics.	Delivering bioactive molecules, modulating immune responses, and promoting tissue repair.	Further research needed to understand the mechanisms and potential.	2c
Popowski et al. [36]	Exosomes in lung regenerative medicine.	Treating COPD, pulmonary fibrosis, and lung cancer.	Further research needed to understand the mechanisms.	2b
Sanghani et al. [37]	Exosome therapies in ophthalmology.	Treating eye diseases and promoting tissue repair.	Need for further research on the mechanisms and efficacy.	2c
Kavya et al. [39]	Therapeutic applications of exosomes.	Potential in treating cancer, cardiovascular disease, neurological disorders, and infectious diseases.	Challenges in standardization and efficacy.	1c
Kost et al. [38]	Exosome therapy in hair regeneration.	Stimulating hair growth and improving follicle function.	Need for standardization in production and purification methods.	2c
Willis et al. [41]	Exosomes in cardiovascular disease therapy.	Challenges in development, heterogeneity, and understanding biological functions.	Need for research on fit-for-purpose potency.	2c
Salarpour et al. [40]	Exosomes and nanoparticle complexes for brain disorders.	Crossing the blood–brain barrier, targeted delivery for Alzheimer’s, Parkinson’s, and stroke.	Further research needed to understand potential.	1b

**Table 4:** Key points and classification of the of the exosomes in diagnostic and therapeutic tools.

Authors	Focus	Key Points	Conclusion	Evidence Level
Huda et al. [42]	Potential uses of exosomes in biomedical applications.	Diagnostics, targeted drug delivery, and gene therapy.	Challenges and limitations noted.	2b
Sun et al. [43]	Cancer cell-derived exosomes in clinical applications.	Role in cancer development, immune evasion, and targeted delivery.	Challenges and limitations highlighted.	2b
Xu et al. [44]	Exosome-based immunotherapy for cancer treatment.	Delivering therapeutic agents; advantages of targeted delivery.	Challenges in manufacturing and characterization noted.	2c
Dorayappan et al. [51]	Exosomes in ovarian cancer.	Diagnostic and therapeutic tools, modulating immune response.	Potential for improving understanding and treatment of ovarian cancer.	1b
Lorenc et al. [52]	Exosomes as personalized contrast media and theranostics.	Applications in imaging, diagnosis, therapy, and personalized medicine.	Further research needed to understand properties and potential.	5
Codispoti et al. [53]	NANOmetric BIO-banked MSC-derived exosomes (NANOBIOME).	Proprietary method for isolating and preserving exosomes; preclinical studies show promise.	Potential for wide range of therapeutic applications.	2c
Tang et al. [54]	Cancer exosomes: clinical implications and challenges.	Role in progression, metastasis, immune evasion, and therapy vectors.	Challenges in standardization and characterization methods.	5
Tzng et al. [56]	Challenges in exosome-based treatments.	Lack of standardization, scalability, and regulatory approval issues.	Need for improved characterization, tracking, and manufacturing processes.	2b
Nafar et al. [57]	Exosomes in cancer treatment.	Role in development, progression, and therapeutic agents.	Challenges in understanding.	5
